# To the Members of the Maryland State Dental Association and the Washington City Dental Society

**Published:** 1896-01

**Authors:** 


### Editorial, Etc.

TO THE MEMBERS OF THE MARYLAND STATE DENTAL ASSOCIATION AND THE WASHINGTON CITY DENTAL SOCIETY—GREETING:—The most meagre account of the results of the Union Meetings of the Maryland State Dental Association and the Washington City Dental Society, in 1894 and in 1895, would include a marked advance in the prestige of these organizations in the world of Dentistry; an increase of knowledge through the presentation and discussion of papers of a high order on various subjects of enduring interest and the very gratifying reports thereof by the leading Dental Journal; an enhancement of mutual respect and of the professional spirit among those in attendance; the happy renewing of old and the forming of new friendships—this latter alone more than compensating to many their loss of time and expense.

Both organizations unhesitatingly expressed their satisfaction with the outcome of these meetings by charging the undersigned with the duty for arranging for similar meetings in Washington in 1896 and in Baltimore in 1897. In the arrangement of committees for the meeting in 1896, a complete list of which we append hereto, we have acted with consideration for the chief object—the accomplishment of much preparatory work with a view of making this meeting more successful than either of the preceeding. We have had to look over the whole field of probabilities and consider many matters not likely to be thought of by persons who view the scheme partially or from individual standpoints, and it is hoped and expected that not a single declination of assigned duty will be made.

We appeal to your professional pride to put yourself in touch with the general purposes of these meetings, and especially urge you to find your place in the plan of committees and to work with zeal and enthusiasm with your fellow-committeemen and this committee, believing that by such effort and co-operation our next meeting will be made memorable as the grandest achievement of our time-honored associations.

Every one is expected to contribute something, even if it be but an "incident of office practice," and; in order that a program may be printed in due time and this committee enabled to arrange for the orderly presentation of papers, clinics and committee reports, *every contributor, clinician and chairman of committee* SHOULD REPORT BY MARCH 15, 1896, to Dr. B. Holly Smith, Secretary Joint Committee.

*Bear in mind that the full measure of success depends on individual, personal effort*—YOUR EFFORT.

Fraternally,

T. S. WATERS,
756 N. Eutaw St. Baltimore, Md.
WM. A. MILLS,
122 S. Broadway, Baltimore, Md.
B. HOLLY SMITH,
1013 Madison Ave., Baltimore, Md.
WMS. DONNALLY,
1022 14th St., Washington, D. C.
J. H. P. BENSON,
1107 9th St., Washington, D. C.
A. W. SWEENY,
813 Vermont Ave., Washington, D. C.
*Joint Committee of Arrangements.*

COMMITTEES FOR JOINT MEETING OF MARYLAND STATE DENTAL ASSOCIATION AND WASHINGTON CITY DENTAL SOCIETY, 1896.

*Publication and Voluntary Essays.*—A. W. Sweeny, Chairman 813 Vermont Avenue, Washington, D. C.; H. M. Schooley, Metzerott Building, Washington, D. C.; Fanny Hoopes, Cor. Charles and Read Streets, Baltimore, Md.; A. P. Gore, Baltimore, Md.; A. Price, Baltimore, Md.

*Anatomy, Physiology and Histology.*—W. S. Twilley, Chairman, 309 N. Eutaw Street, Baltimore, Md.; C. J. Grieves, 720 N. Howard St., Baltimore, Md.; H. C. Thompson, 1113 Penna. Ave., Washington, D. C.; Jas. H. Harris, 857 N. Eutaw St., Baltimore, Md.; W. E. Diffenderfer, 931 N. Y. Ave., Washington, D. C.

*Operative Dentistry.*—C. M. Gingrich, Chairman, 608 St. Paul St., Baltimore, Md.; C. C. Harris, 857 N. Eutaw St., Baltimore, Md.; R. B. Donaldson, 1309 F. Street, Washington, D. C.; T. H. Davy, 336 N. Charles St., Baltimore, Md.; J. B. Ten Eyck, 1601 O St., Washington, D. C.

*Prosthetic Dentistry.*—J. R. Walton, Chairman, 700 10th St., Washington, D. C.; J. Hall Lewis, 1309 F St,, Washington, D. C. G. M. Smith, 1013 Madison Ave., Baltimore, Md.; F. M. Seebold, 1315 N. Y. Ave., Washington, D. C.; Geo. E. Hardy, 716 Park Ave., Baltimore, Md.

*Dental Education, Literature and Nomenclature.*—F. J. S. Gorgas, Chairman, 845 N. Eutaw St., Baltimore, Md.; Isaac H. Davis, 331 N. Charles St., Baltimore, Md.; W. L. Harban, 1407 N. Y. Ave., Washington, D. C.; A. J. Volck, 338 N. Charles St., Baltimore, Md.; G. L. Hills, 17th and H. Sts., Washington, D. C.

*Pathology and Therapeutics.*—L. C. F. Hugo, Chairman, 808 17th St., Washington, D. C.; D. E. Wiber, 717 11th St., Washington, D. C.; W. A. Mills, 122 S. Broadway, Baltimore, Md.; W. S. Harban, 1342 N. Y. Ave., Washington, D. C.; G. H. Claude, Annapolis, Md.

*Crown and Bridge Work.*—W. B. Finney, Chairman, 815 N. Eutaw St., Baltimore, Md.; T. S. Waters, 756 N. Eutaw St., Baltimore, Md.; G. B. Welsh, 1344 G. St., Washington, D. C.; S. G. Davis, 607 13th St., Washington, D. C.; F. W. Parker, 4th and E. Capitol Sts., Washington, D. C.

*Orthodontia and Dental Appliances.*—M. F. Finley, Chairman, 1928 I St., Washington, D. C.; Chas. B. Munson, 1324 N. Y. Ave.; Washington, D. C.; David Genese, 621 Calhoun St., Baltimore, Md.; S. C. Pennington, 327 N. Charles St., Baltimore, Md.; W. W. Bruce, 1307 W. Fayette, St., Baltimore, Md.

*Oral Surgery.*—P. E. Sasscer, Chairman, Waldorf, Md.; M. G. Sykes, Ellicott City, Md.; A. D. Cobey, 700 10th St., Washington, D. C.; Lewis Buffet, Easton, Md.; A. J. Brown, 14th St., near K, Washington, D. C.

*Anæsthetics.*—L. L. Harban, Chairman, 1342 N. Y. Ave., Washington, D. C.; R. W. Talbot, 1111 F. St., Washington, D. C.; E. E. Cruzen, Cumberland, Md.; C. T. Lindsey, 1209 31st St., Washington, D. C.; C. E. Duck, 112 W. Mulberry St., Baltimore, Md.

*Hygiene.*—H. W. Lakin, Chairman, Boonesboro, Md.; C. S. Grindall, 421 N. Charles St., Baltimore, Md.; W. M. Ash, 470 Florida Ave., Washington, D. C.; W. N. Coogan, 719 11th St., Washington, D. C.; C. W. Appler, G St., near 14th St., Washington, D. C.

*Dental Legislation.*—H. B. Noble, Chairman, 1324 N. Y. Ave., Washington, D. C.; J. L. Wolf, 1313 N. Y. Ave., Washington, D. C.; A. C. McCurdy, Towson, Md.; Richard Grady, 720 N. Howard St., Baltimore, Md.; Ed. Nelson, Frederick, Md.

